# Getting an Evolutionary Handle on Life after Reproduction

**DOI:** 10.1371/journal.pbio.0040016

**Published:** 2005-12-27

When Richard Dawkins famously called organisms “throwaway survival machines” that exist solely to preserve the genes that made them, critics balked at the specter of genetic determinism. But Dawkins' selfish genes derive straight from classical evolutionary theory that says life exists to reproduce, and that natural selection should act on any traits that increase reproductive success. Since many animals live beyond their fertile years, biologists have searched for evolutionary clues to this extended lifespan. What role, if any, does natural selection play in the evolution of the postreproductive lifespan?

For natural selection to shape the twilight years, postreproductive females should contribute to the fitness of their offspring or relatives, a hypothesis called the “grandmother effect.” Such contributions require that organisms spawn helpless offspring or live in extended groups where postreproductive females help raise the young. Though many mammals, including lions and baboons, rear dependent young and operate within complex social groups, studies have found no evidence of a granny effect, and females mostly live just long enough to care for their last born. For nonsocial animals that spawn independent young, extended lifespan is associated with good nutrition and the absence of disease and predators. While historical records and demographic analyses offer support for an adaptive granny effect in humans—which some biologists have offered as a possible explanation for the existence of menopause—few studies have experimentally tested for signs of selection in the evolution of a postreproductive lifespan. [Fig pbio-0040016-g001]

**Figure pbio-0040016-g001:**
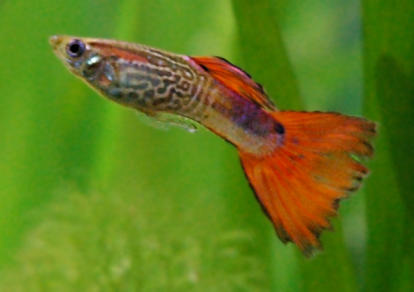
Analysis of the causes of variation in post-reproductive lifespan indicates that the evolution of lifespan in guppies is due to selection during their reproductive stage

In a new study, David Reznick, Michael Bryant, and Donna Holmes expand on their ongoing investigations of the life history of guppies confronting different predatory threats in Trinidad. Individuals facing different mortality threats should evolve different adaptations in their life histories, such as age at first reproduction, investment in reproduction, and patterns of senescence, including declines in reproduction. Since guppies are livebearers that provide no postnatal maternal care, Reznick et al. predicted the populations would show no differences in postreproductive lifespan—which is what they found. Though overall lifespan varied among the populations, these variations stemmed from differences in time allotted only to reproduction. Postreproductive lifespan, in contrast, showed no signs of being under selection, and appeared to be what the authors called a “random add-on at the end of the life history.” Random or not, this is the first demonstration of a postreproductive lifespan in fish.

Reznick et al. raised a second-generation laboratory brood of wild guppies taken from high- and low-predation streams at two locations in the mountains of Trinidad. (The high-predation sites harbor predators that frequently prey on guppies. Low-predation sites are found in the same streams, above waterfalls that exclude predators but not guppies.) A high- and low-predation site was sampled from each site, and feeding was manipulated to reflect food availability in the wild (fish in low-predation environments typically eat less and weigh less than fish in high-predation environments). Females were mated once a week until they produced offspring, and were mated again after each brood (when copulation is most likely).

The authors measured growth rate, body size, interbrood interval, and litters per lifetime for each population, and divided each individual's lifespan into age at first birth, reproductive phase, and postreproductive lifespan. Guppies from high-predation localities gave birth sooner than those from low-predation sites; they also reproduced over a longer period and were much older when they stopped reproducing. To estimate postreproductive lifespan, Reznick et al. determined whether the time between last birth and death significantly exceeded the time needed to spawn another litter (calculated as a threshold, since interbrood intervals varied for each individual). About 60% of individuals lived beyond the time they would have been expected to produce another brood. While the authors found no differences in the probability that any particular group would enjoy an extended postreproductive lifespan or that an individual would stop reproducing before dying, they did find that the probability of experiencing an extended postreproductive lifespan increased along with the length of reproductive lifespan. Thus, even though postreproductive lifespan has no direct effect on fitness, it is linked to a component of life history that does.

Altogether, these results provide the first experimental confirmation that evolution works selectively on those aspects of life history that directly affect fitness. These findings also refute the suggestion that fish may experience little or no reproductive senescence based on evidence that they continue to produce eggs as adults. It's an open question whether postreproductive lifespan can influence fitness enough to be under selection. But in a field dominated by investigations into the origins of human menopause and extended lifespan, the authors make a strong case for using experimental comparative analyses of other species to gain an evolutionary perspective on the human condition. *—Liza Gross*

